# Evaluation of *Pediococcus pentosaceus* SP2 as Starter Culture on Sourdough Bread Making

**DOI:** 10.3390/foods9010077

**Published:** 2020-01-10

**Authors:** Stavros Plessas, Ioanna Mantzourani, Argyro Bekatorou

**Affiliations:** 1 Laboratory of Food Processing, Department of Agriculture Development, Democritus University of Thrace, 68200 Orestiada, Greece; imantzou@agro.duth.gr; 2 Department of Chemistry, University of Patras, 26500 Patras, Greece; abekatorou@upatras.gr

**Keywords:** *Pediococcus pentosaceus* SP2, sourdough bread starter, acidity, volatile compounds, microbial spoilage

## Abstract

In the present study, a novel *Pediococcus pentosaceus* SP2 strain, recently isolated from kefir grains, was evaluated as a starter culture in sourdough bread making. The novel starter was applied in fresh, freeze-dried, and freeze-dried immobilized (on wheat bran) form. The type of culture (fresh, freeze-dried, immobilized cells) influenced the bread characteristics. Specifically, the application of freeze-dried immobilized cells led to higher total titratable acidity (TTA) values (9.81 mL NaOH N/10), and the produced bread presented higher resistance to mold and rope spoilage. Moreover, the produced sourdough breads were significantly better in terms of pH, TTA, organic acids content, and resistance to mold and rope spoilage, compared to breads made with a commercial, wild microbiota, sourdough. The organic acids content was also significantly higher than the commercial sourdough sample (2.93 g/kg lactic acid; 1.01 g/kg acetic acid). Determination of volatile compounds through solid-phase microextraction (SPME) gas chromatography/mass spectrometry (GC/MS) analysis and sensorial assessments indicated no significant differences between the tested sourdough breads.

## 1. Introduction

The use of sourdough is well established as a natural, free of synthetic additives, method of bread making. Specifically, the advantages of sourdough are the production of breads with (i) higher nutritional value (minerals, free amino acids, and protein bioavailability), (ii) enhanced palatability, (iii) superior organoleptic characteristics (increased production of desirable volatile compounds), and (iv) increased shelf life (lower staling rate, high resistance to rope and mold spoilage) [1]. Sourdough is a food matrix with a complex microbial load that can be composed by lactic acid bacteria (LAB), acetic acid bacteria, and yeasts [2]. Consequently, its microbiological stability is critical and should be controlled in industrial production. A way to simplify this matter is the application of pure starters that will prevail over other species present in the dough. In addition, the control of fermentation conditions (e.g., pH, fermentation time and temperature) is also very significant because it can favor the growth of the starter culture [3,4].

Selected LAB are often used in sourdough starter culture preparations, because they are naturally present at high populations in the natural sourdough microflora and due to many advantages they offer in breads, such as enhanced flavor profile, higher preservation times, and increased nutritional value. Moreover, the potential health benefits (e.g., probiotic properties and antagonism against food-borne pathogens and spoilage organisms), has increased the consumer preference for foods containing LAB. Many such examples of LAB applications in sourdough preparations have been reported in the scientific literature [5,6,7,8,9,10].

Kefir grains have also been applied as mixed starters for sourdough bread making [11,12,13]. The kefir grains microbiota consist mainly of *Lactobacillus*, *Streptococcus*, *Lactococcus* species, and yeasts, among a variety of other species, which coexist in perfect symbiosis [14]. Likewise, kefir grains are considered a valuable source of beneficial microbes (mainly LAB) that can exhibit either probiotic properties or other functional properties of technological interest [11,15,16].

On the other hand, the food industry requires ready-to-use microbial starters that can be preserved for long periods, maintaining their functionality at high levels. Preservation methods, which are well established as being practical for industrial applications, are sub-cultivation, freezing, and drying [17]. However, preservation by drying seems to be the most appropriate, since it is a low-cost method, and because it produces compact, easily stored, and relatively lightweight final products [18]. Freeze-drying is also a widespread drying technique for microorganisms, which offers several advantages such as high stability and high viability of the cultures during storage [18,19].

Therefore, the efficiency of *P. pentosaceus* SP2, in free-form, freeze-dried form, and immobilized on wheat bran and freeze-dried form, as a starter culture in sourdough bread making is sought in the present study. Typical bread parameters were assessed, such as physicochemical characteristics, resistance to spoilage, flavor-related compounds, and consumer acceptance. The ultimate target will be to evaluate the technological performance of *P. pentosaceus* SP2 for application in probiotic food production in general, which is a significant prerequisite for candidate probiotic strains [20,21]. In this study, sourdough was selected as the food matrix because: (i) *P. pentosaceus* has been identified in the natural sourdough microbiota [22], and (ii) some *P. pentosaceus* strains seem to have antimicrobial properties against microbial bread spoilage [23]. For these reasons, *P. pentosaceus* SP2 was evaluated in this study as a potential starter culture in sourdough bread making. Moreover, the culture was also immobilized on wheat bran as a means to enhance its viability since immobilization has been previously shown to be a useful technique for the survival of cells during processing and storage [24]. 

## 2. Materials and Methods

### 2.1. Microorganisms

*P. pentosaceus* SP2, recently isolated from kefir grains [16], was grown in MRS (De Man, Rogosa and Sharpe) broth (Fluka, Buchs, Switzerland) at 37 °C for 24 h. Suitable amounts of harvested cell biomass (approximately 4% *w*/*w* wet weight basis) were then obtained and used for sourdough bread making. Baker’s yeast was a commercial *Saccharomyces cerevisiae* strain (S.I. Lesaffre, France), supplied in the form of compressed blocks.

### 2.2. Cell Immobilization 

*P. pentosaceus* SP2 was immobilized on wheat bran, which was supplied by a local cereal processing company (Orestiada, Greece). The immobilization procedure included the mixing of 0.5 g of harvested cell mass with 5 g of wheat bran in 500 mL MRS broth and incubating at 37 °C for 48 h. Afterwards, the immobilized cells were washed twice with Ringers solution ¼ strength for the removal of free cells [24,25].

### 2.3. Freeze-Drying 

Free and immobilized cells of *P. pentosaceus* SP2 were freeze-dried overnight on a freeze-drying system (FreeZone 4.5, Labconco, Kansas City, MO, USA). Subsequently, they were applied as starter cultures for sourdough bread production [25].

### 2.4. Determination of Cell Counts

The determination of viable cell counts of freshly harvested, freeze-dried, and freeze-dried/immobilized *P. pentosaceus* SP2 was done as follows: 1 g of each sample was homogenized in 9 mL of phosphate buffer (1.25 mL of 0.25 M solution of KH_2_PO_4_ per litre of distilled water). The suspension was serially diluted, plated on MRS agar (Fluka, Buchs, Switzerland) and incubated at 37 °C for 48–72 h. The cell counts were expressed as log cfu/g of freshly harvested *P. pentosaceus* SP2 cells or of wheat bran in the case of immobilized and freeze-dried/immobilized cells [25].

The determination of viable cell counts of LAB and yeasts in the sourdoughs was carried out in a similar manner. Specifically, 20 g of sourdough were homogenized in 200 mL of phosphate buffer. The suspension was serially diluted, and LAB was determined on MRS agar (Fluka, Buchs, Switzerland) after incubation at 37 °C for 48–72 h and yeasts were determined on malt agar (Fluka, Buchs, Switzerland) after incubation at 30 °C for 2 days [25].

### 2.5. Sourdough Bread Making 

Commercial white flour was used for bread making (Hellenic Biscuit CO S.A., Athens, Greece), with the following composition (% *w*/*w*): Protein 11.0, carbohydrates 72.0, fat 1.5, fiber 2.2 and moisture 12.0. Mixing of the ingredients was performed mechanically, and the dough was molded manually in 1.5 L baking pans.

Three mother sponges were prepared by mixing 300 g wheat flour, 160 mL tap water, and 1% *w*/*w* (on flour basis) of (i) *P. pentosaceus* SP2 culture, or (ii) freeze-dried *P. pentosaceus* SP2 culture, or (iii) immobilized freeze-dried *P. pentosaceus* SP2 for 15 min. All the sponges were incubated at 30 °C for 24 h. 

Sourdoughs were prepared by mixing, for 15 min, 250 g of the above-fermented mother sponges with 300 g wheat flour and 160 mL tap water, followed by incubation at 30 °C for 24 h. The sourdoughs were coded as Fresh SP2 (prepared with fresh *P. pentosaceus* SP2), Freeze-dried SP2 (prepared with freeze-dried *P. pentosaceus* SP2), and Immobilized SP2 (prepared with immobilized freeze-dried *P. pentosaceus* SP2). Subsequently, 3 respective sourdough breads were produced containing 30% *w*/*w* (on flour basis) of these sourdoughs (bread with Fresh SP2 sourdough, bread with Freeze-dried SP2 sourdough, and bread with Immobilized SP2 sourdough). The doughs of all the breads contained 150 g of sourdough, 500 g wheat flour, 270 mL tap water, and 4 g salt. In all cases, an amount of 1% *w*/*w* (on flour basis) of pressed baker’s yeast was also added as a leavening agent. All doughs were fermented at 30 °C for 2 h, proofed at 40 °C for 60 min and baked at 230 °C for approximately 40 min [4]. 

In addition, trials were carried out for comparison using traditional (wild microbiota) sourdough provided by a local bakery (sourdough coded as C). This sourdough is used 2–3 times per week and is, respectively, refreshed in order to obtain the appropriate acidity and viability of LAB. The produced bread (bread sample C) contained 30% (on flour basis) of a traditional wild microbiota sourdough. The recipe and the procedure followed was the same as described above for the *P. pentosaceus* SP2 sourdoughs. All trials were carried out in triplicate.

### 2.6. Organic Acids Analysis

Lactic, acetic, formic, propionic, n-valeric, and caproic acid were determined by ion-exchange liquid chromatography, as described previously by [25]. Determinations of organic acid concentrations were carried out by means of standard curves.

### 2.7. Determination of pH and Total Titratable Acidity

The pH and total titratable acidity (TTA) values of sourdough bread samples were determined as described previously [4]. The TTA was expressed as the volume (mL) of NaOH N/10 consumed.

### 2.8. Determination of Specific Loaf Volume

Loaf volume was measured by the rapeseed displacement method. The specific loaf volume was calculated as mL/g [25].

### 2.9. Analysis of Flavor Volatiles

Determination of volatile compounds was done by gas chromatography/mass spectrometry (GC/MS) analysis. Initially, volatile compounds were isolated by the headspace solid-phase microextraction (SPME) sampling technique, as described before [13]. The identification of volatile compounds was carried out through comparison with standard compounds (Sigma-Aldrich, Saint Louis, MO, USA) and MS data with those in NIST107, NIST21, and SZTERP libraries. 4-methyl-2-pentanol (Sigma-Aldrich) diluted in pure ethanol was used as the internal standard (IS) at various concentrations (4, 40, and 400μg/g of sample) for the semi-quantitative analysis of volatiles. The quantification of the volatile compounds was made by dividing the peak areas of the compounds of interest by the peak area of the IS and multiplying this ratio by the initial concentration of the IS. All assays were carried out in triplicate.

### 2.10. Rope and Mould Spoilage Observation

Sourdough breads made with Fresh SP2 sourdough, with Freeze-dried SP2 sourdough, and with Immobilized SP2 sourdough, as well as with the commercial sourdough (C), were examined for rope and mold spoilage. Specifically, sourdough bread samples of similar shape and size were cut from the same loaf of bread and stored at room temperature. The appearance of rope spoilage and mold spoilage was evaluated macroscopically as described before [4]. All assays were carried out in triplicate.

### 2.11. Sensory Evaluation

Sourdough breads made with Fresh SP2 sourdough, with Freeze-dried SP2 sourdough and with Immobilized SP2 sourdough, as well as with the commercial sourdough (C), were evaluated at a local bakery through a blind sensory evaluation test immediately after their production. Specifically, 15 randomly untrained testers (consumers) evaluated the breads providing scores between 0 (unacceptable) and 10 (exceptional) for attributes of flavor, taste, and overall quality such as volume, texture, and color. At least 3 samples were delivered to each tester. A scoring scale with 3 categories was applied: Class 1 related to high-quality bread without any off-odor or off-flavor, class 2 related to bread with slight off-odors or off-flavors and class 3 related to the bread of unacceptable quality. The results were expressed as average scores plus standard deviations [25]. 

### 2.12. Statistical Analysis

The effects of the different sourdough starters on the physicochemical characteristics of the breads, the volatile flavour compounds, and the scores of the sensory tests were analyzed by ANOVA followed by Duncan’s post hoc multiple range test to extract the specific differences between the various treatments. The statistical analysis was performed by using IMB SPSS v20 (IBM Corp., Armonk, NY, USA) at an alpha level of 5%.

## 3. Results and Discussion

### 3.1. Sourdough Bread Quality Characteristics

In general, no statistically significant differences were observed among the produced bread samples (*p* > 0.05) regarding specific loaf volumes and n-valeric and caproic acid levels. All other characteristics of the sourdough breads produced with any form of *P. pentosaceus* SP2 sourdough, were better compared to the bread produced with the commercial sourdough (C). Specifically, *P. pentosaceus* SP2 breads had statistically significant differences regarding pH values compared to the commercial sourdough breads (C) (Table 1). 

**Figure 1:**
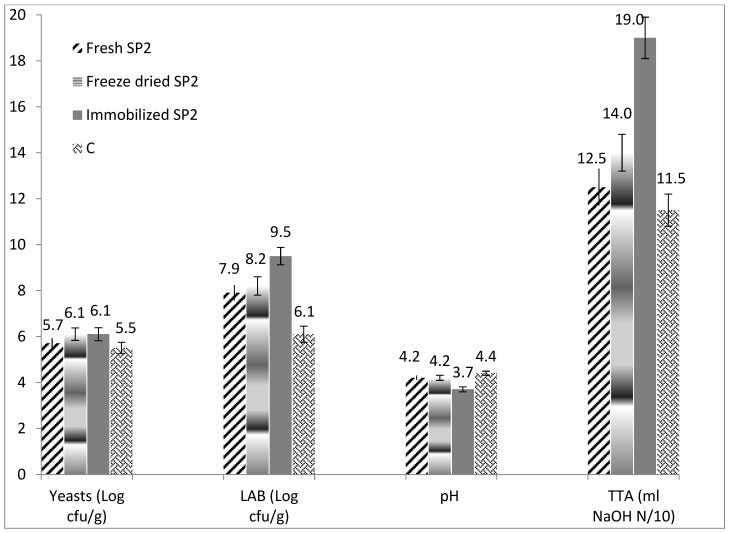
Viable cell counts (log cfu/g) of yeasts and lactic acid bacteria (LAB), total titratable acidity (TTA), and pH values of the sourdoughs prepared with frsh *P. pentosaceus* SP2 cells (Fresh SP2), with freeze-dried *P. pentosaceus* SP2 cells (Freeze-dried SP2), with immobilized freeze-dried *P. pentosaceus* SP2 cells (Immobilized SP2), and commercial sourdough with wild microbiota (C), after incubation at 30 °C for 24 h.

A higher TTA value (9.81 mL NaOH N/10), higher lactic acid (2.93 g/kg) and propionic acid content (0.08 g/kg) (*p* < 0.05), were determined in the sourdough bread made with Immobilized SP2 sourdough compared to all the other bread samples (Table 1). This may be explained by the fact that this sourdough had the lowest pH value (3.7) and highest TTA (19 mL NaOH N/10) than all the other sourdoughs revealing its high potential for bread acidification. Another explanation for this significant difference is that sourdough prepared with immobilized freeze-dried *P. pentosaceus* SP2, contained statistically higher viable cell counts of LAB (9.5 log cfu/g) compared to all the other sourdoughs (Figure 1). This result is quite interesting, since the initial viable cell counts of *P. pentosaceus* SP2 in the three different sourdoughs after their preparation were approximately the same (8.08 ± 0.12, 8.04 ± 0.09, and 8.04 ± 0.11 log cfu/g for the fresh *P. pentosaceus* SP2 sourdough, the freeze-dried SP2 sourdough, and the freeze-dried immobilized SP2 sourdough, respectively), while those of LAB in the commercial sourdough were 8.07 ± 0.11 log cfu/g (on wet weight basis). The initial cell counts of the biocatalysts used to prepare the sourdoughs were 8.23 ± 0.06, 8.18 ± 0.05, and 8.11 ± 0.8 log cfu/g for the fresh *P. pentosaceus* SP2 culture, the freeze-dried culture, and the freeze-dried immobilized biocatalyst, respectively. It should be underlined that the sourdough used in bakeries is appropriately activated and refreshed many times before its application for sourdough bread making. This is why, although no starter culture was added in the commercial sourdough, the initial cell counts for LAB were at about the same levels as those of the other tested sourdoughs. Likewise, the addition of starter culture in the sourdough and the application of immobilization clearly enhanced the viability of *P. pentosaceus* SP2 in the sourdoughs after incubation at 30 °C for 24 h, as shown in Figure 1. Specifically, wheat bran (a cereal processing by-product) that was used as the immobilization carrier is considered as a prebiotic substrate which enhances the viability of probiotic bacteria and can be incorporated in novel functional foods [24]. 

### 3.2. Volatile Compounds

The composition of headspace volatile compounds of the produced breads was analyzed by SPME GC/MS, and the results are presented in Table 2. In total, 33 compounds were detected in all sourdough breads containing *P. pentosaceus* SP2, and 24 in the bread made by the commercial sourdough (C). Most of these compounds (especially those with low odor threshold values) are well known to affect bread flavor (sourdough and non-sourdough), such as benzaldehyde, heptanol, 2-phenylethyl acetate, hexanal, 2-methylbutanal, 2-phenylethanol, 1-octen-3-ol, 2-nonenal, furfural, etc., and their contribution to bread flavor has been widely reviewed [26]. The most important observation in this case, was the identification of more esters (10) in the case of the sourdough breads that contained *P. pentosaceus* SP2, compared to the commercial sourdough bread (5 compounds), which is a common observation for most fermented foods and is expected to affect flavor since esters are usually associated with pleasant fruity and flowery notes.

### 3.3. Appearance of Spoilage

**Figure 2:**
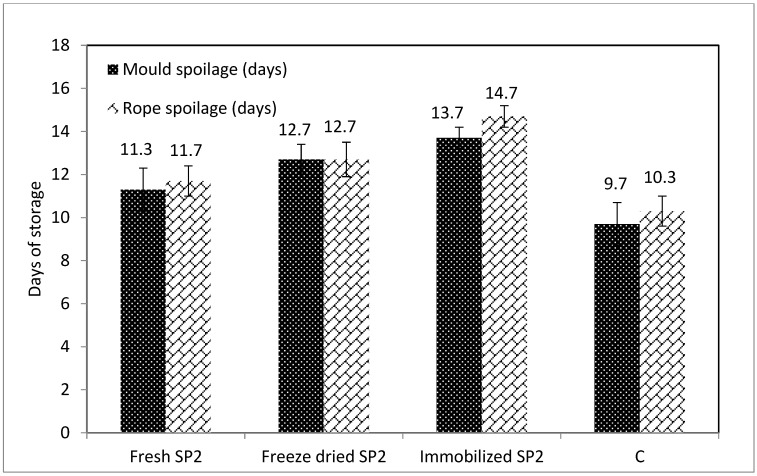
Resistance against rope and mold spoilage of breads made with sourdoughs containing *P. pentosaceus* SP2 cells (Fresh SP2), freeze-dried *P. pentosaceus* SP2 cells (Freeze-dried SP2), immobilized freeze-dried *P. pentosaceus* SP2 cells (Immobilized SP2), as well as with the commercial sourdough (C).

It is widely recognized that the microbial spoilage of bread determines its shelf life and causes considerable economic losses. Therefore, the role of LAB in sourdough bread spoilage is a widely studied topic. The published work has shown that organic acids produced by LAB species present strong antimicrobial activity in bread, depending on the sensitivity of the individual spoilage organisms, while they affect the activity of baker’s yeast, the rising of the dough, as well as the texture of the bread [4,5,6,7,8,23,25,27,28,29,30]. The appearance of mold and rope spoilage in all sourdough bread samples was monitored through daily macroscopic observations, which are depicted in Figure 2.

Regarding mold spoilage, it seems that the sourdough bread made with immobilized SP2 sourdough was more resistant (*p* < 0.05) compared to the other samples, since spoilage was observable after the 13th day of storage. The sourdough bread made with Fresh SP2 sourdough developed observable spoilage after the 11th day, sourdough bread made with Freeze -dried SP2 sourdough after the 12th day, while the commercial sourdough bread (C) developed spoilage after the 9th day. The better resistance to spoilage of the sourdough bread made with immobilized SP2 sourdough may be attributed to its higher TTA and organic acid content, as also discussed in previous studies. Specifically, it has been reported that acetic acid presents higher antifungal activity than other organic acids produced by LAB, and it can also have a desirable effect on bread flavor at a certain concentration range [23].

In addition to their antifungal capacity, the organic acids and other compounds produced by LAB, also present effective antibacterial activities. This may explain the delayed (*p* < 0.05) development of rope spoilage in the sourdough bread made with Immobilized SP2 sourdough (after the 14th day), i.e., 2–4 days later than the other bread samples. This is in agreement with a recent study reporting that the use in sourdough bread making of a novel *L. paracasei* K5 strain delayed rope spoilage [4]. The antimicrobial effect of *P. pentosaceus* has also been previously shown in meat applications [31]. In addition, it has been reported that in sourdough fermented with *Lactobacillus plantarum* and *P. pentosaceus*, rope spoilage was delayed effectively, provided that the pH of the sourdoughs was below 4.0, and TTA was higher than 12 [27].

### 3.4. Consumer Acceptability

The results of the customer-oriented sensory evaluation of the produced sourdough breads are presented in Table 3. In general, the evaluation did not reveal significant differences among the different bread types. Likewise, the fact that the proposed starter culture did not receive lower scores compared to the commercial sourdough that was applied weekly in the bakery can be considered a positive outcome. In addition, even though wheat bran could affect the texture and volume of the bread negatively, as highlighted in previous studies [24], this was not observed by the evaluators.

## 4. Conclusions

Control of bread spoilage by natural means is significant from economic, food safety, as well as consumer acceptance points of view. Specifically, the microbiological stability of sourdough is important and should be controlled in industrial bread production. This can be achieved by using selected, efficient LAB species. The aim of this study was to assess the technological performance in sourdough bread making of a novel *P. pentosaceus* SP2 strain, recently isolated from kefir grains. The results showed that *P. pentosaceus* SP2 can be efficiently used as a sourdough starter. The produced breads were better in terms of acidity, organic acid content, and resistance to spoilage, compared to commercial sourdough bread (wild microbiota) prepared under the same conditions. In addition, the novel starter was more effective in the immobilized, freeze-dried form (on wheat bran), while immobilization enhanced its viability in sourdoughs. These findings indicate the potential for commercialization of the *P. pentosaceus* SP2 strain in the form of dry, lightweight, and reservable immobilized preparations for industrial purposes. Future work is needed to evaluate if the novel strain is able to produce particular metabolites such as exopolysaccharides and bacteriocins, as well as its potential for probiotic food production [28].

## Figures and Tables

**Table 1:** Physicochemical characteristics of breads made with sourdough containing *P. pentosaceus* SP2 cells (Fresh SP2), freeze-dried *P. pentosaceus* SP2 cells (Freeze-dried SP2), immobilized freeze-dried *P. pentosaceus* SP2 cells (Immobilized SP2), as well as with the commercial sourdough (C).

Type of Sourdough Applied	pH	TTA	Specific Loaf Volume	Organic Acids (g/kg bread)						
		(mL NaOH N/10)	(mL/g)	Lactic	Acetic	Formic	Propionic	n-Valeric	Caproic	Total av.
Fresh SP2	4.45 ± 0.02 ^b^	8.29 ± 0.10 ^b^	2.48 ± 0.13 ^a^	2.64 ± 0.05 ^b^	0.99 ± 0.04 ^a^	0.07 ± 0.01 ^b^	0.05 ± 0.01 ^b^	0.05 ± 0.01 ^a^	0.04 ± 0.01 ^a^	3.84
Freeze-dried SP2	4.43 ± 0.03 ^b^	8.78 ± 0.12 ^a^	2.36 ± 0.10 ^a^	2.71 ± 0.04 ^b^	0.96 ± 0.05 ^a^	0.09 ± 0.01 ^b^	0.05 ± 0.01 ^b^	0.05 ± 0.01 ^a^	0.05 ± 0.01 ^a^	3.91
Immobilized SP2	4.46 ± 0.03 ^b^	9.81 ± 0.10 ^a^	2.39 ± 0.11 ^a^	2.93 ± 0.05 ^a^	0.96 ± 0.04 ^a^	0.12 ± 0.01 ^a^	0.08 ± 0.01 ^a^	0.07 ± 0.01 ^a^	0.05 ± 0.01 ^a^	4.21
C	4.75 ± 0.04 ^a^	7.23 ± 0.10 ^c^	2.46 ± 0.09 ^a^	2.10 ± 0.11 ^c^	1.01 ± 0.04 ^a^	0.08 ± 0.01 ^b^	0.04 ± 0.01 ^b^	0.04 ± 0.01 ^a^	0.03 ± 0.01 ^a^	3.30

TTA: Total Titratable Acidity; Tr: Traces (<0.01 g/kg). Different superscript letters in a column indicate statistically significant differences (ANOVA, Duncan’s multiple range test, *p* < 0.05).

**Table 2:** SPME GC/MS analysis (semi-quantitative) of flavor-related compounds (μg/g) extracted from breads made with sourdoughs containing *P. pentosaceus* SP2 cells (Fresh SP2), freeze-dried *P. pentosaceus* SP2 cells (Freeze-dried SP2), immobilized freeze-dried *P. pentosaceus* SP2 cells (Immobilized SP2), as well as with the commercial sourdough (C).

KI	Compound	RI	Concentration (μg/g)			
			Type of Sourdough Applied			
			Fresh SP2	Freeze-Dried SP2	Immobilized SP2	C
*Alcohols*						
832	Ethanol	A	4.38 ± 0.10 ^a^	4.21 ± 0.12 ^a^	4.33 ± 0.12 ^a^	4.58 ± 0.10 ^a^
1012	Isobutyl alcohol	A	0.19 ± 0.02 ^b^	0.15 ± 0.01 ^b^	0.23 ± 0.03 ^a^	0.06 ± 0.01 ^c^
1120	Isoamyl alcohol	A	0.28 ± 0.09 ^b^	0.18 ± 0.02 ^b^	0.29 ± 0.04 ^a^	0.12 ± 0.02 ^c^
1160	Butan-1-ol	A	0.14 ± 0.02 ^b^	0.14 ± 0.01 ^d^	0.22 ± 0.03 ^a^	0.21 ± 0.03 ^c^
1230	Pentan-1-ol	B	0.12 ± 0.01 ^a^	0.15 ± 0.02 ^c^	0.14 ± 0.01 ^a^	0.10 ± 0.01 ^b^
1257	Hexan-1-ol	A	0.14 ± 0.03 ^b^	0.13 ± 0.03 ^b^	0.19 ± 0.04 ^a^	0.16 ± 0.02 ^b^
1435	Heptan-1-ol	B	0.04 ± 0.01 ^b^	0.03 ± 0.01 ^a^	0.04 ± 0.01 ^a^	nd
1466	Octan-1-ol	A	0.10 ± 0.01 ^a^	0.12 ± 0.02 ^a^	0.12 ± 0.01 ^a^	nd
1480	Heptan-2-ol	A	0.04 ± 0.01 ^b^	0.04 ± 0.01 ^a^	0.08 ± 0.01 ^a^	nd
1540	1-Octen-3-ol	B	0.10 ± 0.01 ^a^	0.15 ± 0.02 ^b^	0.18 ± 0.02 ^a^	nd
1670	Benzylalcohol	A	0.11 ± 0.02 ^a^	0.11 ± 0.01 ^b^	0.18 ± 0.02 ^a^	0.13 ± 0.01 ^b^
1812	2-Phenylethanol	A	0.25 ± 0.02 ^a^	0.24 ± 0.03 ^b^	0.29 ± 0.02 ^a^	0.25 ± 0.02 ^b^
*Esters*						
<800	Ethyl acetate	A	0.19 ± 0.04 ^a^	0.18 ± 0.03 ^b^	0.19 ± 0.04 ^b^	0.15 ± 0.02 ^b^
1107	Butyl acetate	A	0.05 ± 0.01 ^a^	0.06 ± 0.01 ^b^	0.06 ± 0.01 ^b^	0.07 ± 0.01 ^b^
1162	Hexyl acetate	B	0.07 ± 0.01 ^a^	0.06 ± 0.01 ^a^	0.05 ± 0.01 ^a^	0.05 ± 0.01 ^b^
1250	Ethyl pentanoate	B	0.09 ± 0.01 ^a^	0.07 ± 0.01 ^a^	0.07 ± 0.01 ^a^	0.03 ± 0.01 ^b^
1395	Ethyl hexanoate	B	0.08 ± 0.01 ^a^	0.05 ± 0.01 ^a^	0.05 ± 0.01 ^a^	nd
1438	Ethyl octanoate	B	0.08 ± 0.02 ^a^	0.05 ± 0.01 ^a^	0.06 ± 0.01 ^a^	nd
1590	Isobutyl acetate	B	0.12 ± 0.01 ^a^	0.10 ± 0.01 ^a^	0.09 ± 0.01 ^a^	nd
1848	Ethyl dodecanoate	B	0.05 ± 0.01 ^a^	0.05 ± 0.01 ^a^	0.04 ± 0.01 ^a^	nd
1850	2-Phenylethyl acetate	B	0.04 ± 0.01 ^a^	0.05 ± 0.01 ^a^	0.05 ± 0.01 ^a^	nd
2410	Ethyl octadecanoate	B	0.04 ± 0.01 ^b^	0.05 ± 0.01 ^b^	0.05 ± 0.01 ^b^	Tr
*Carbonyl compounds*						
<800	Acetaldehyde	B	0.12 ± 0.02 ^a^	0.11 ± 0.04 ^b^	0.10 ± 0.01 ^b^	0.07 ± 0.01 ^c^
812	2-Methylbutanal	B	0.08 ± 0.01 ^b^	0.07 ± 0.01 ^a^	0.06 ± 0.01 ^a^	0.03 ± 0.01 ^b^
986	3-Methylbutanal	A	0.06 ± 0.02 ^c^	0.06 ± 0.02 ^a^	0.04 ± 0.01 ^b^	0.05 ± 0.01 ^c^
1002	Hexanal	A	0.07 ± 0.01 ^b^	0.09 ± 0.01 ^a^	0.05 ± 0.01 ^c^	0.05 ± 0.01 ^c^
1080	Heptanal	A	Tr	Tr	Tr	Tr
1334	Furfural	A	0.25 ± 0.03 ^a^	0.27 ± 0.04 ^a^	0.20 ± 0.01 a	0.15 ± 0.02 ^a^
1358	Nonanal	B	0.05 ± 0.01 ^a^	0.05 ± 0.01 ^c^	0.05 ± 0.01 ^b^	Tr
1448	γ-Butyrolactone	B	0.89 ± 0.15 ^a^	1.25 ± 0.05 ^b^	1.33 ± 0.02 ^c^	0.69 ± 0.10 ^c^
1458	Benzaldehyde	A	0.28 ± 0.03 ^a^	0.29 ± 0.03 ^b^	0.22 ± 0.03 ^c^	0.21 ± 0.03 ^b^
1541	2-Nonenal	B	0.13 ± 0.05 ^a^	Tr	Tr	0.09 ± 0.02 ^b^
1582	5-Methylfurfural	B	0.12 ± 0.02 ^a^	0.10 ± 0.01 ^b^	0.07 ± 0.01 ^b^	0.07 ± 0.01 ^b^

KI: Kovats Index; RI: Reliability of identification. A: Positive identification by MS data and retention times and those of standard compounds; B: Positive identification by MS data only; Tr: Compound present at <0.01 μg/g bread (traces); nd: Not detected. Different superscript letters in a row indicate statistically significant differences (ANOVA, Duncan’s multiple range test, *p* < 0.05).

**Table 3:** Scores of the customer-oriented sensory evaluation of the breads made with sourdoughs prepared with *P. pentosaceus* SP2 cells in free-form (Fresh SP2), freeze-dried form (Freeze-dried SP2), and immobilized freeze-dried form (Immobilized SP2), as well as with the commercial sourdough (C).

Type of Sourdough	Flavor	Taste	Appearance	Overall Quality
Fresh SP2	8.9 ± 0.1 ^a^	8.3 ± 0.1 ^a^	8.1 ± 0.1 ^a^	8.5 ± 0.1 ^a^
Freeze^-^dried SP2	8.8 ± 0.1 ^a^	8.4 ± 0.1 ^a^	8.0 ± 0.1 ^a^	8.4 ± 0.1 ^a^
Immobilized SP2	8.8 ± 0.2 ^a^	8.2 ± 0.2 ^a^	8.1 ± 0.2 ^a^	8.6 ± 0.2 ^a^
C	8.8 ± 0.2 ^a^	8.3 ± 0.1 ^a^	8.1 ± 0.2 ^a^	8.5 ± 0.2 ^a^

Different superscript letters in a row indicate statistically significant differences (ANOVA, Duncan’s multiple range test, *p* < 0.05.
